# De Novo Graves’ Disease in a 10-Year-Old Girl Receiving Gonadotropin-Releasing Hormone Agonist Therapy for Idiopathic Central Precocious Puberty: A Case Report

**DOI:** 10.7759/cureus.85231

**Published:** 2025-06-02

**Authors:** Yoshimi Fujita, Kenta Horimukai, Mika Kiyohara, Noriko Takahata

**Affiliations:** 1 Department of Pediatrics, Jikei University Katsushika Medical Center, Tokyo, JPN

**Keywords:** autoimmune thyroid diseases, central precocious puberty, gonadotropin-releasing hormone agonists, graves' disease, leuprolide

## Abstract

We present a case of Graves' disease in a 10-year-old girl with a family history of thyroid disease, which may represent a significant risk factor. The patient developed this condition while receiving gonadotropin-releasing hormone (GnRH) agonist therapy for idiopathic central precocious puberty. The patient presented with headache and vomiting during leuprorelin acetate treatment. Thyroid function tests revealed suppressed thyroid-stimulating hormone (TSH) levels accompanied by elevated free triiodothyronine (FT3) and free thyroxine (FT4) concentrations and positive TSH receptor antibodies (TRAb). Thyroid ultrasonography demonstrated diffuse glandular enlargement with increased vascularity, consistent with Graves' disease. Subsequently, GnRH agonist therapy was discontinued, and thiamazole treatment resulted in normalized thyroid function. Given the rarity of Graves' disease in the pediatric population, this case suggests a potential contributory role of GnRH agonist therapy in its onset. Current evidence suggests that GnRH agonists precipitate autoimmune thyroid disorders predominantly through two pathways: (i) a sustained hypoestrogenism-driven immune rebound state and (ii) direct GnRH-mediated lymphocyte activation. These mechanisms, combined with our patient's family history, highlight the potential importance of thyroid function monitoring in children receiving long-term GnRH agonist therapy, particularly those with a similar genetic predisposition.

## Introduction

Central precocious puberty (CPP) is an endocrine disorder characterized by the early onset of secondary sexual characteristics due to the premature activation of the hypothalamic-pituitary-gonadal (HPG) axis [1]. Its prevalence in children ranges from 0.01% to 0.02% [2]. More than 50% of children with autoimmune thyroid diseases, including Hashimoto's thyroiditis and Graves' disease, have a positive family history of autoimmune thyroid disorders, emphasizing the critical role of genetic susceptibility in disease development [3]. The prevalence is particularly elevated during puberty, with approximately 78% of pediatric autoimmune thyroid disease cases occurring during pubertal stages, underscoring the importance of hormonal influences in disease manifestation [3]. CPP can compromise final adult height and lead to various psychosocial challenges, highlighting the importance of early diagnosis and timely intervention.

CPP results from the premature gonadotropin-releasing hormone (GnRH)-dependent activation of the HPG axis. GnRH agonists, which are the first-line therapy, initially cause a transient increase in luteinizing hormone (LH) and follicle-stimulating hormone (FSH) levels before downregulating pituitary GnRH receptors. This suppression of hormones effectively delays pubertal progression and bone maturation [4,5].

GnRH agonists are generally well tolerated, with most adverse events being transient and related to acute fluctuations in sex hormone levels. However, autoimmune complications, though rare, are increasingly recognized. Recent reports have highlighted unexpected alterations in thyroid function, ranging from subclinical thyroid-stimulating hormone (TSH) elevation to overt thyrotoxicosis [6,7]. Although several pediatric cases of autoimmune thyroiditis during GnRH agonist therapy have been documented [5,8], progression to overt Graves’ disease remains exceedingly rare, with no unequivocal pediatric cases reported to date. Here, we describe a well-documented pediatric case of de novo Graves’ disease arising during long-term GnRH agonist therapy and discuss plausible immunological mechanisms.

## Case presentation

A 10-year-old Japanese female diagnosed with idiopathic central precocious puberty (ICPP) presented to our hospital with a three-day history of headache and vomiting. She had been receiving GnRH agonist therapy for 13 months, initiated at nine years and eight months of age. Baseline thyroid function tests were obtained before starting GnRH agonist therapy, revealing mildly suppressed TSH (0.37 µIU/mL; reference range: 0.67-4.5 µIU/mL [9]), while FT3 (3.22 pg/mL) and FT4 (1.19 ng/dL) were within normal ranges. Thyroid auto-antibody titers (TgAb, TPOAb, TRAb) were, however, not assessed at baseline, which remains a limitation of this report. Thyroid function tests revealed severe biochemical hyperthyroidism, with TSH suppressed to <0.01 μIU/mL and marked elevations in both FT3 >25.0 pg/mL (reference range: 3.1-4.9 pg/mL [9]) and FT4 >8.0 ng/dL (reference range: 0.96-1.6 ng/dL [9]). These findings prompted admission for the management of hyperthyroidism. Her medical history was limited to ICPP, with no additional pharmacotherapy. Although detailed social information was unavailable due to her institutional placement, a family history of thyroid dysfunction on her mother's side was noted.

Diagnosis and treatment of central precocious puberty

The patient initially presented at eight years and nine months of age. Her clinical history revealed premature breast development before the age of eight years, accelerated linear growth (height: 134.0 cm; +2.4 SD), and advanced bone age by approximately 2 years and 10 months, as assessed by left-hand radiography using the Tanner-Whitehouse two method, along with elevated sex hormone levels consistent with ICPP. Following an informed discussion with her guardians, a conservative observational approach was initially selected due to the absence of immediate psychosocial concerns or risks to final adult stature. However, at nine years and six months of age, she experienced menarche, thereby fulfilling the Japanese diagnostic criteria for CPP [10].

Due to her difficulty in managing menstrual changes, GnRH agonist therapy was initiated. After 13 months of continuous treatment, at the age of 10 years and 9 months, the patient developed symptoms of hyperthyroidism. An initial dose of 36 μg/kg effectively suppressed estradiol (E2) levels and gonadotropin secretion, resulting in cessation of menses. The dosage was adjusted based on serial LH measurements, as shown in Table 1. Over the course of treatment, 13 injections were administered at four-week intervals, with dosages of 36 μg/kg (four administrations), 45 μg/kg (four administrations), and 62 μg/kg (five administrations).

**Table 1:** Timeline of GnRH agonist therapy and endocrine parameters Following initiation of GnRH agonist therapy, LH levels decreased from a clearly pubertal value (23.3 mIU/mL) to consistently suppressed values below the pubertal threshold (from 1.2 to 0.7 mIU/mL), with concurrent estradiol suppression (from 206.0 pg/mL to <10 pg/mL). Clinical improvement, including cessation of menses, confirmed therapeutic efficacy. Thyroid autoantibody testing was not performed at these time points. Baseline thyroid function tests are reported in the text. LH, luteinizing hormone; E2, estradiol; GnRH, gonadotropin-releasing hormone

	9 years 6 months	9 years 10 months	10 years 2 months	10 years 6 months
Treatment status	Baseline (pre-GnRH agonist)	+2 months	+6 months	+10 months
LH (mIU/mL)	23.3	1.2	0.8	0.7
Estradiol (pg/mL)	206.0	<10	<10	<10
GnRH agonist (μg/kg)	0	36	45	62

Diagnosis of hyperthyroidism

At the time of hyperthyroidism evaluation, the patient's anthropometric measurements were as follows: height 153.2 cm (+1.8 SD), weight 45.4 kg (+1.5 SD), and a documented weight loss of 2 kg over the preceding two months. The patient did not exhibit diaphoresis or tremors. Vital signs revealed tachycardia with a pulse rate of 113 beats per minute, a body temperature of 37.1°C, and elevated blood pressure of 126/68 mmHg. Physical examination showed a diffusely enlarged, non-tender thyroid gland (World Health Organization [WHO] grade 1) in the anterior neck, without discrete nodularity, warmth, exophthalmos, or edema.

**Figure 1:**
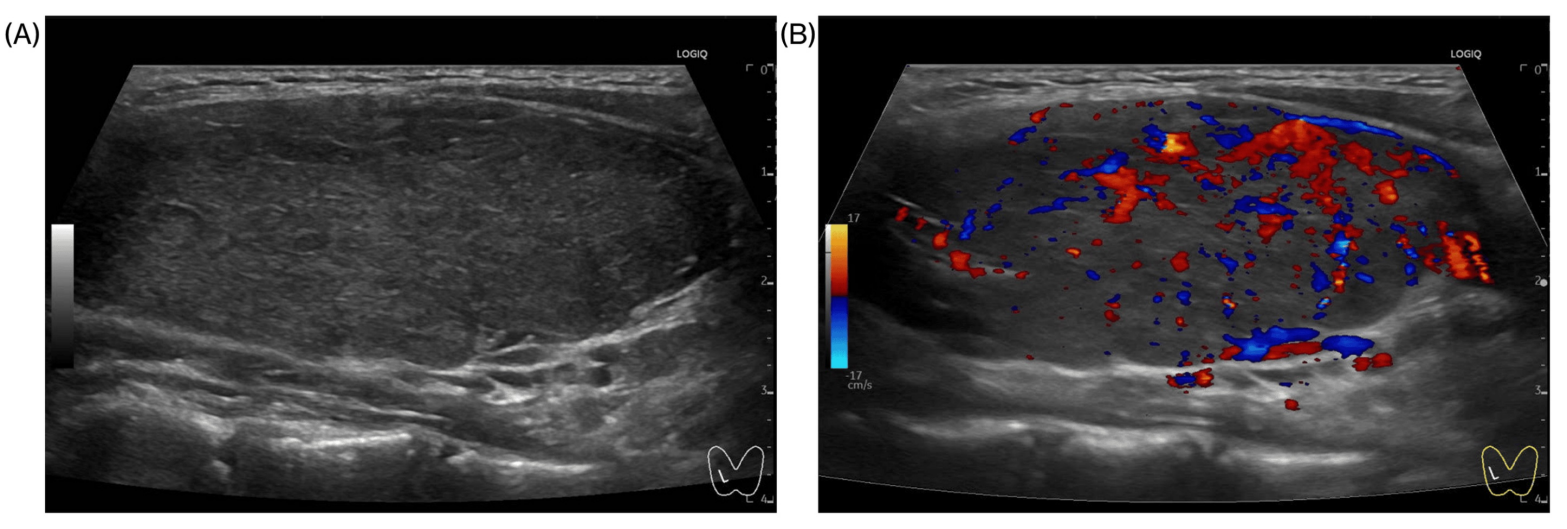
Ultrasonographic images of Graves' disease Longitudinal ultrasonographic images of the thyroid gland of a 10-year-old girl with Graves' disease during GnRH agonist therapy. (A) B-mode ultrasonography revealed diffuse thyroid enlargement with homogeneous parenchymal echogenicity. (B) Color Doppler imaging demonstrates marked hypervascularity with a “thyroid inferno” pattern, a characteristic finding of Graves' disease. GnRH, gonadotropin-releasing hormone

Serological studies demonstrated markedly elevated thyroid-stimulating antibody levels (5940%) and TSH receptor antibodies (TRAb) (23.8 IU/L). Thyroid ultrasonography confirmed a diffusely enlarged gland with increased vascularity and no nodules (Figure 1). Based on the clinical features of thyrotoxicosis, diffuse thyroid enlargement, elevated FT3 and FT4 levels with suppressed TSH, and positive TRAb, a diagnosis of Graves' disease was established, in accordance with the Japanese guidelines for childhood-onset Graves' disease [11].

Treatment of hyperthyroidism

**Figure 2:**
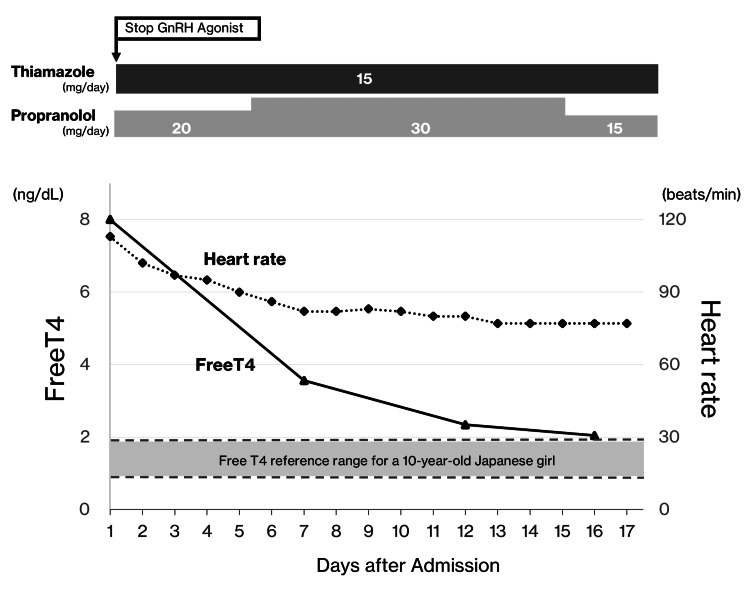
Clinical response to treatment discontinuation and antithyroid therapy After the GnRH agonist therapy was discontinued, thiamazole was initiated at 15 mg/day for Graves' disease, along with propranolol at 20 mg/day to control tachycardia. The patient’s tachycardia resolved by the seventh day of hospitalization, and by the 16th day, the FT4 levels normalized (as illustrated in the graph). The FT3 levels returned to normal over the same period (data not shown). No severe complications were encountered. GnRH, gonadotropin-releasing hormone

Considering the possibility that GnRH agonist therapy might have influenced the onset or progression of Graves' disease, and after discussing the risks and benefits with her guardians, we discontinued GnRH agonist therapy, particularly since the patient was now managing menses adequately and there were no concerns regarding her predicted adult height. For the management of Graves' disease, thiamazole was initiated at 15 mg daily, and propranolol was concurrently administered at 20 mg daily to control tachycardia. The patient was monitored closely for the potential development of thyroid storm and thiamazole-related adverse events (e.g., fever, hepatic dysfunction, and agranulocytosis). Notably, her tachycardia resolved by the seventh day of hospitalization, and FT3 and FT4 levels normalized by the 16th day. No episodes of thyroid storm or other serious complications were observed (Figure 2).

During the 12-month follow-up period after initiating thiamazole, the dosage was gradually tapered, and thyroid hormone levels were serially monitored. The patient is currently maintained on 5 mg thiamazole every other day, with no recurrence of hyperthyroidism. Although no adverse reactions to thiamazole have been noted to date, long-term surveillance continues to monitor for potential relapses.

## Discussion

To our knowledge, this report describes one of the earliest documented pediatric cases of de novo Graves' disease emerging during prolonged GnRH agonist therapy for CPP, rigorously confirmed by a board-certified pediatric endocrinologist using diagnostic criteria consistent with current international guidelines [10,11]. Although causality cannot be established from a single case, the close temporal association and underlying genetic predisposition suggest that GnRH agonist therapy may have acted as a proximate trigger. Emerging evidence has linked GnRH agonists to thyroid dysfunction. Adult case reports have described the onset of Graves’ disease following treatment with buserelin and/or goserelin, or during controlled ovarian stimulation (COS), which involves pharmacological evaluation of FSH and human chorionic gonadotropin (hCG) excess [6,12,13].

Because COS entails supraphysiological gonadotropin levels, the “specificity spillover” mechanism - cross-activation of the TSH receptor by LH, FSH, or hCG - may explain thyrotoxicosis in that setting. However, under long-acting GnRH agonist therapy, LH and FSH are chronically suppressed following the initial flareup phase. Therefore, spillover is biologically implausible in CPP and was not considered the principal mechanism in our patient.

These two complementary pathways warrant further investigation. First, the concept of hypoestrogenism-driven immune rebound is biologically plausible. During pregnancy, immune responses are suppressed under high estrogen levels, but they rapidly recover in the postpartum period, enhancing humoral immunity - possibly via increased autoantibody production from CD5⁺ B cells - and may trigger autoimmune diseases such as Graves' disease [14]. Similarly, the initiation of GnRH agonist therapy induces a marked reduction in circulating estrogen levels, creating a hormonal shift analogous to the transition from pregnancy to the postpartum period. This abrupt estrogen withdrawal may likewise unmask or accelerate immune activity, resulting in a loss of tolerance and increased TRAb production. In our patient, estradiol levels remained consistently below 10 pg/mL throughout treatment, supporting the hypothesis that estrogen deficiency contributed to the development of Graves' disease.

Second, direct GnRH-mediated immunostimulation may have contributed to this outcome. Functional GnRH and gonadotropin receptors are expressed in T and B lymphocytes, and GnRH is also produced locally by these cells, suggesting an autocrine role [15,16]. In vitro and animal studies have demonstrated that both GnRH and gonadotropins enhance lymphocyte proliferation and promote the release of pro-inflammatory cytokines [15,16]. These direct effects could synergize with immune rebound to unmask latent autoimmunity and drive the production of TRAb.

Genetic susceptibility increases the risk of autoimmune thyroid disease. Most reported cases of GnRH agonist-associated thyroid dysfunction have occurred in individuals with positive thyroid autoantibodies or a family history of autoimmune diseases [6,17]. In our case, a maternal history of thyroid dysfunction reinforces this pattern and underscores the importance of baseline risk stratification. Given our patient's baseline mildly suppressed TSH (0.37 µIU/mL), more frequent monitoring with thyroid autoantibodies could have detected the progression to autoimmune hyperthyroidism earlier. Early detection of thyroid dysfunction through systematic monitoring could potentially allow for timely intervention and better outcomes. This case underscores the critical importance of establishing comprehensive baseline thyroid assessment (including autoantibodies) and implementing periodic surveillance, particularly in children with a genetic predisposition to autoimmune thyroid disease.

Although this report is limited to a single case and previous studies are largely retrospective, the clinical implications are clear. A significant limitation of our case is the absence of baseline thyroid autoantibody measurements (TPOAb, TgAb, TRAb) prior to GnRH agonist initiation. While baseline thyroid function tests revealed mildly suppressed TSH with normal FT3 and FT4 levels, the lack of autoantibody assessment prevented detection of any pre-existing autoimmune thyroid activity. Our findings suggest that children with the following risk factors should undergo particularly careful monitoring during GnRH agonist therapy: (1) a positive family history of autoimmune thyroid disease, (2) the presence of thyroid autoantibodies at baseline, and (3) a prolonged duration of therapy. Initial screening should include TSH, FT3, FT4, and thyroid autoantibodies (anti-TPO, TgAb, and TRAb). While the optimal frequency of follow-up testing remains to be established through prospective studies, regular clinical assessment for symptoms of thyroid dysfunction is essential.

In summary, our findings support a model in which sustained estrogen deprivation, combined with direct GnRH-driven immunomodulation, rather than gonadotropin spillover, triggers the onset of Graves’ disease in genetically susceptible children undergoing GnRH agonist therapy. Heightened clinical vigilance will allow clinicians to balance the established benefits of GnRH agonist therapy against potential autoimmune risks and to intervene promptly when thyroid dysfunction arises.

## Conclusions

This case highlights a potential association between GnRH agonist therapy and the emergence of Graves' disease in genetically predisposed pediatric patients, though causality cannot be established from a single case report. The primary clinical takeaway is the importance of comprehensive baseline thyroid assessment (including thyroid autoantibodies) and periodic monitoring during GnRH agonist therapy, particularly in children with a family history of thyroid disease. Prospective multicenter studies are warranted to establish incidence rates, clarify pathophysiological mechanisms, and determine evidence-based surveillance intervals.
